# Hematopoietic stem cells on the crossroad between purinergic signaling and innate immunity

**DOI:** 10.1007/s11302-023-09943-0

**Published:** 2023-05-15

**Authors:** Stephanie Franczak, Henning Ulrich, Mariusz Z. Ratajczak

**Affiliations:** 1 Laboratory of Regenerative Medicine, Medical University of Warsaw, Warsaw, Poland; 2 Stem Cell Institute at James Graham Brown Cancer Center, University of Louisville, 500 S. Floyd Street, Rm. 107, Louisville, KY 40202 USA; 3 Department of Biochemistry, University of Sao Paulo, Sao Paulo, Brazil

**Keywords:** Purinergic signaling, Complement, Complosome, Innate immunity, NLRP3 inflammasome, Nox2, ROS, Stem cell homing and engraftment, Stem cell metabolism, Hematopoiesis

## Abstract

Hematopoiesis is regulated by several mediators such as peptide-based growth factors, cytokines, and chemokines, whose biological effects have been studied for many years. However, several other mediators have been identified recently that affect the fate of hematopoietic stem/progenitor cells (HSPC) as well as non-hematopoietic cells in the bone marrow microenvironment. These new mediators comprise members of purinergic signaling pathways and are active mediators of the soluble arm of innate immunity, the complement cascade (ComC). In this review, we will discuss the coordinated effects of these pathways in regulating the biology of HSPC. Importantly, both purinergic signaling and the ComC are activated in stress situations and interact with specific receptors expressed on HSPC. Evidence has accumulated indicating that some of the purinergic as well as ComC receptors could also be activated intracellularly by intrinsically expressed ligands. To support this recent evidence, our work indicates that the major mediator of purinergic signaling, adenosine triphosphate, and the cleavage product of the fifth component of the ComC (C5), C5a anaphylatoxin, can activate their corresponding receptors expressed on the outer mitochondrial membrane in an autocrine manner. We will also discuss recent evidence that these responses, mediated by purinergic signaling and the ComC network, are coordinated by activation of the nicotinamide adenine dinucleotide phosphate (NADPH) oxidase 2 - reactive oxygen species - NLR family pyrin domain containing 3 (NLRP3) inflammasome (Nox2-ROS-NLRP3) axis.

## Introduction

The fate of hematopoietic stem/progenitor cells (HSPC) giving rise to different hematopoietic/lymphopoietic lineages is regulated in steady state conditions by signals mediated by a plethora of growth factors, cytokines, chemokines, and bioactive lipids [1, 2]. However, HSPC may respond to various stressors beyond homeostatic regulation, and these other mediators play an important role [3–6]. This situation occurs during infections, tissue organ injuries, and in response to myeloablative therapy as seen after conditioning for hematopoietic transplantation or after administration of pro-mobilizing factors that promote egress of HSPC from the bone marrow (BM) into peripheral blood (PB) [3, 7–9].

While cortisol and catecholamines are known stress hormones regulating the HSPC response to stress, mounting evidence indicates involvement of purinergic signaling mediators and active products of the soluble arm of innate immunity, the complement cascade (ComC) [3, 10]. To support this evidence, recent research demonstrated that purinergic signaling ligands, particularly extracellular adenosine triphosphate (eATP) and its metabolite extracellular adenosine (eAdo), oppositely regulate several aspects of HSPC biology [11]. It is well known that eATP, a danger/damage associated molecular pattern (DAMP) molecule or alarmin, is released into the extracellular space from stressed/damaged cells [6]. On the other hand, stress activates the ComC, and mediators released in response to activation of complement C3 and C5 factors, such as C3a or C5a anaphylatoxins, play an important role in orchestrating the response of HSPC to external or internal stressors [3, 4, 12, 13].

**Fig. 1:**
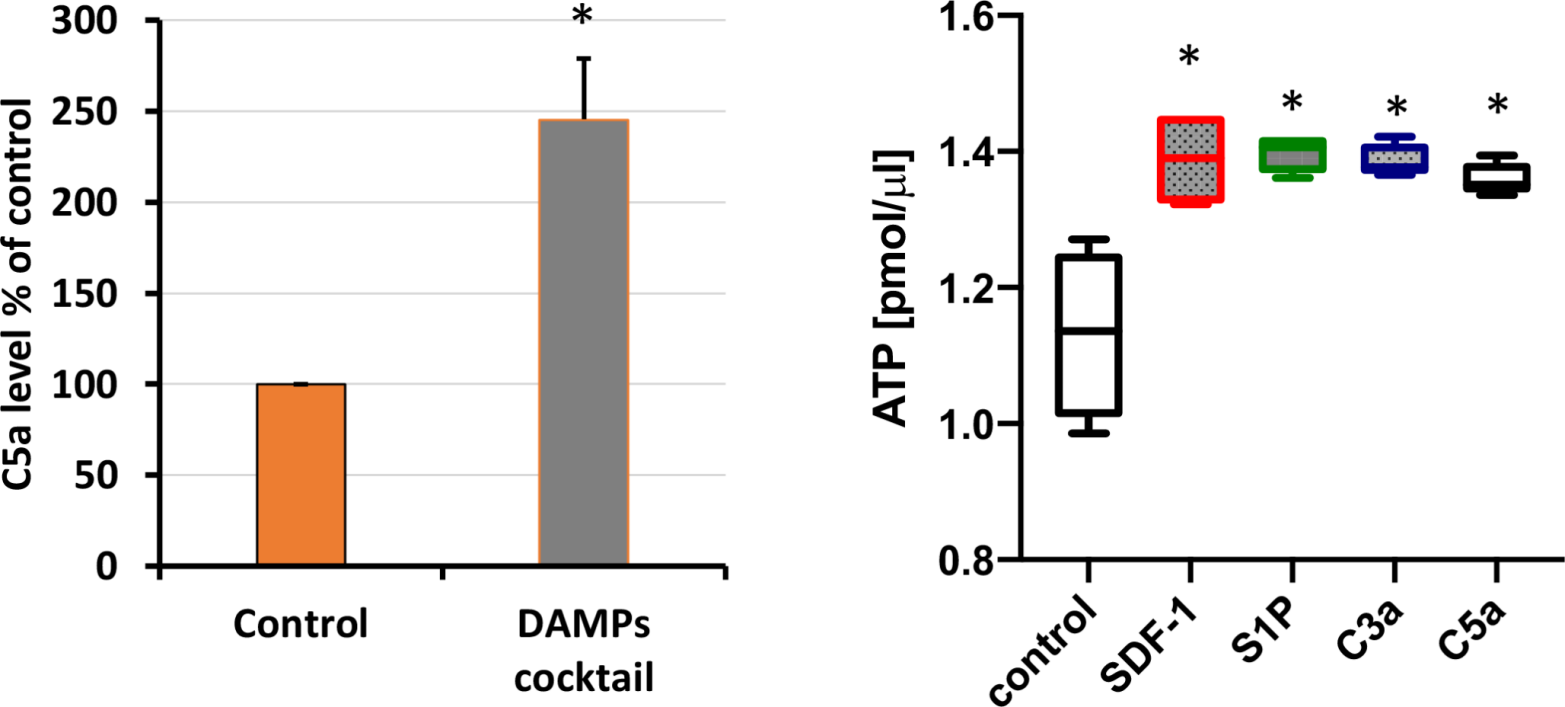
Mutual activation of the ComC and purinergic signaling in response to stress. Left Panel. Activation of the complement cascade and release of C5a in mice after intravenous infusion of DAMPs (ATP [3 mg/kg; Sigma-Aldrich, St. Louis, MO, USA] + HMGB1 [1.5 µg; Sino Biological, Beijing, China] + S100A9 [2 µg; Sino Biological]). C5a level was measured in PB using ELISA. *p < 0.001. (This panel was published by the authors in Oncotarget (permission obtained for reproduction from reference [40]). **Right Panel**. Intravenous infusion in mice of C3a (1 mg/ml) and C5a (140 ng/ml), similar to SDF-1 (5 ng/ml) and S1P (0.1 mM), elevates the level of eATP in a murine peripheral blood. Measurement of eATP by ELISA was performed 3 h after infusion. *p < 0.001

Important to this review is the fact that release of alarmins, including eATP, from stressed/damaged cells activates the ComC; conversely, activation of the ComC in PB elevates the level of circulating eATP in PB (Fig. 1). This indicates an interplay between both danger sensing pathways and their mutual, supportive interaction in regulating the biology of HSPC. This interplay occurs at the level of specific receptors expressed on the outer cell membrane of HSPC. However, evidence has accumulated that both purinergic and ComC receptors are also expressed intracellularly and could become activated intrinsically by specific ligands [14–19]. To support this evidence, recent findings indicate that the major mediator of purinergic signaling, adenosine triphosphate (ATP), and the cleavage product of the fifth component of the ComC (C5), C5a anaphylatoxin, can activate the P2 × 7 receptor [20] and C5aR receptor [14–19], respectively, expressed on the outer mitochondrial membrane in an autocrine manner. This new data provides insight into a novel level of autocrine regulation of the stress response.

### Purinergic signaling as a novel regulator of hematopoiesis

Purinergic signaling is a primordial form of extracellular signaling mediated by extracellular nucleotides, including the above-mentioned purine, eATP, and its nucleoside metabolite, eAdo [21]. While eATP engages several ionotropic P2X and G-protein coupled P2Y receptors, eAdo stimulates the family of G-protein coupled P1 receptors. In addition to eATP and eAdo, there are other rare signaling extracellular nucleotides including some pyrimidines like UTP, UDP, or UDP-glucose [21]. The P2X ionotropic channel receptor family stimulated exclusively by eATP consists of seven members (P2 × 1, 2, 3, 4, 5, 6, and 7), whereas the P2Y family responding to ATP, adenosine diphosphate (ADP), uridine triphosphate (UTP), uridine diphosphate (UDP), or UDP-glucose includes a total of eight receptors (P2Y1, 2, 4, 6, 11, 12, 13, and 14), and the P1 family activated by eAdo comprises four subtypes (A1, A2A, A2B, and A3) [21].

Our group investigated the role of purinergic signaling in normal HSPC in detail. First, we confirmed that murine and human HSPC express mRNA for all the purinergic receptors, suggesting that hematopoiesis is regulated by this ancient regulatory network [11, 22]. Based on mRNA analysis data, we became curious as to which of these receptors are important in regulating hematopoiesis. We also inquired into an explanation for the potential redundancy in signaling of many of these receptors expressed by HSPC. First, we focused on P2X ionotropic receptors; human cells expressed mRNA for all seven of these receptors, and murine HSPCs, while also expressing mRNA for all seven receptors, expressed P2 × 5 at a very low level [21, 22]. To study the role of these receptors in the biology of HSPC, we utilized available mutant knock-out (KO) mice (P2 × 4-KO and P2 × 7-KO) and specific inhibitors of the P2 × 1 and P2 × 3 receptors [22–24]. The absence of functional P2 × 1, P2 × 3, P2 × 4, and P2 × 7 receptors on HSPC resulted in defective chemotactic migration to major BM-derived chemoattractants, including a-chemokine stromal derived factor-1 (SDF-1) and bioactive phosphosphingolipid - sphingosine-1 phosphate (S1P) [9, 25]. Defective expression of these receptors resulted in poor trafficking of HSPC, as seen after pharmacological mobilization using cytokine granulocyte colony stimulating factor and C-X-C chemokine receptor type 4 (CXCR4) antagonist small molecule AMD3100 (Plerixafor) [22–24]. On the other hand, lack or blockage of these receptors on HSPCs or BM microenvironment cells resulted in poor homing and engraftment of transplanted murine BM cells in the marrow of the transplant recipients [22–24]. These experiments revealed that, despite a visible defect in cell migration after inhibition of one of these receptors, there exists a redundancy in their function/signaling; specifically, lack of one of these receptors was partially rescued by signaling from other receptors from the P2X family [22]. Such redundancy is characteristic of old regulatory pathways. One of the common intracellular pathways regulated by P2X receptors is activation of the NLRP3 inflammasome, an intracellular pattern recognition receptor [26, 27]. eATP and eAdo have opposite effects on NLRP3 inflammasome activation, which will be discussed later in this review [11]. Intriguing evidence emerged that one of the P2X ionotropic receptors, the P2 × 7 subtype, is expressed and functional on the mitochondrial outer membrane [20]. Whether other purinergic receptors are also intracellularly expressed requires further research. This observation could prompt an exciting new area of investigation on intracellular intracrine regulation by purinergic signaling.

### Innate immunity and its role in regulating hematopoiesis

All hematopoietic cells, including lymphocytes, are developmentally related as they share a common stem cell precursor. As a result, they share some common receptors and respond to similar stimuli [10]. While lymphocytes become specified into T- and B-cell lineages and are responsible for so-called acquired immunity, HSPC give rise to several cell types that form the cellular arm of inborn or innate immunity [12, 13]. These cells comprise phagocytes, mast cells, basophils, eosinophils, and dendritic cells. Innate immunity cells, similar to HSPC, are highly responsive to extrinsic and intrinsic stressors, trauma, and toxic and infectious agents [12, 13]. Depending on stressor type, duration, and strength, they respond by proliferation and differentiation.

Cells belonging to innate and acquired immunity, similar to HSPC, respond to mediators of ComC activation the central soluble arm of innate immunity [12, 13]. The most important ComC active cleavage fragments are C3a and C5a anaphylatoxins and the C5b-C9 membrane attack complex (MAC) [12, 13]. The ComC senses danger signal-related changes in the cell microenvironment and orchestrates responses from innate and acquired immune cells. This ancient sensing system becomes activated by three pathways known as the (i) classical, (ii) mannan binding lectin, and (iii) alternative pathway. All these pathways respond to “danger signals” that could be (i) pathogen associated molecular patterns (PAMP), molecules of infectious microorganism origin or (ii) DAMP, molecules released upon non-infectious sterile inflammation [6]. Both PAMP and DAMP activate several receptors that are expressed on outer cell membranes or in the cytosol [5, 27, 28].

DAMP are the primary mediators of sterile inflammation and are released from stressed cells into extracellular space. They modulate the response of HSPC to stress and may originate from the nucleus, cytosol, mitochondria or extracellular matrix [6]. Active ComC cleavage fragments and DAMP trigger the state of sterile inflammation in hematopoietic tissues as seen, for example, during mobilization of HSPC or after conditioning of hematopoietic transplant recipients by myeloablative therapy. DAMP are sensed by a set of cell surface- and cytosol-expressed pattern recognition receptors, including the family of NOD-like receptors (NLR), such as the NLRP3 inflammasome, and Toll-like receptors (TLR) [5, 27, 28]. While TLR are expressed on the outer cell membrane and in the cytosol, the NLRP3 inflammasome is expressed only in the cytosol [27]. Importantly, the NLRP3 inflammasome becomes activated in response to cell stimulation by P2X receptors, activation of ComC cleavage fragment receptors including C3a and C5a, and the soluble non-lytic signaling MAC [12, 13, 27, 28].

For many years, it was accepted that ComC proteins are synthesized exclusively in the liver and are released into circulating PB, where they become activated in a cascade-dependent manner in response to danger signals [12, 13]. Recent research has demonstrated that ComC proteins are also expressed in other extrahepatic locations, such as in lymphocytes [15–17]. These ComC elements that are expressed in lymphocytes are functional and have been termed “complosome” [15–17]. Interestingly, our recent studies demonstrated the presence of functional “complosome” in murine and human HSPC as well [19, 27]. Therefore, HSPC are not only exposed to liver-derived ComC cleavage fragments circulating in PB, but also response the intrinsic cell-expressed complosome network [19, 27]. In addition to HSPC, complosome is also expressed in non-hematopoietic cells in the BM microenvironment [19, 27].

It is well known that C3a and C5a, ComC active anaphylatoxins circulating in PB, are strong chemotactic factors for innate immunity cells (e.g., granulocytes) [12, 13, 29]. However, we reported that, in contrast to leukocytes, they do not chemoattract HSPC [29]. Despite this fact, mice that are C3- or C5-deficient are poor mobilizers and engraft poorly with bone marrow mononuclear cells from normal, wild type mice [25, 29–31]. This data suggests that HSPC respond to stimulation by C3a and C5a through activation of the NLRP3 inflammasome, an important priming factor for cell responsiveness to crucial BM chemoattractants (e.g., SDF-1 or S1P). Both anaphylatoxins also interact with BM non-hematopoietic microenvironment cells and, in a NLRP3 inflammasome-dependent manner, facilitate homing and engraftment of transplanted BM donor cells [26].

### Activation of the Nox2-ROS-NLRP3 inflammasome axis on the crossroad between purinergic and ComC signaling

**Fig. 2:**
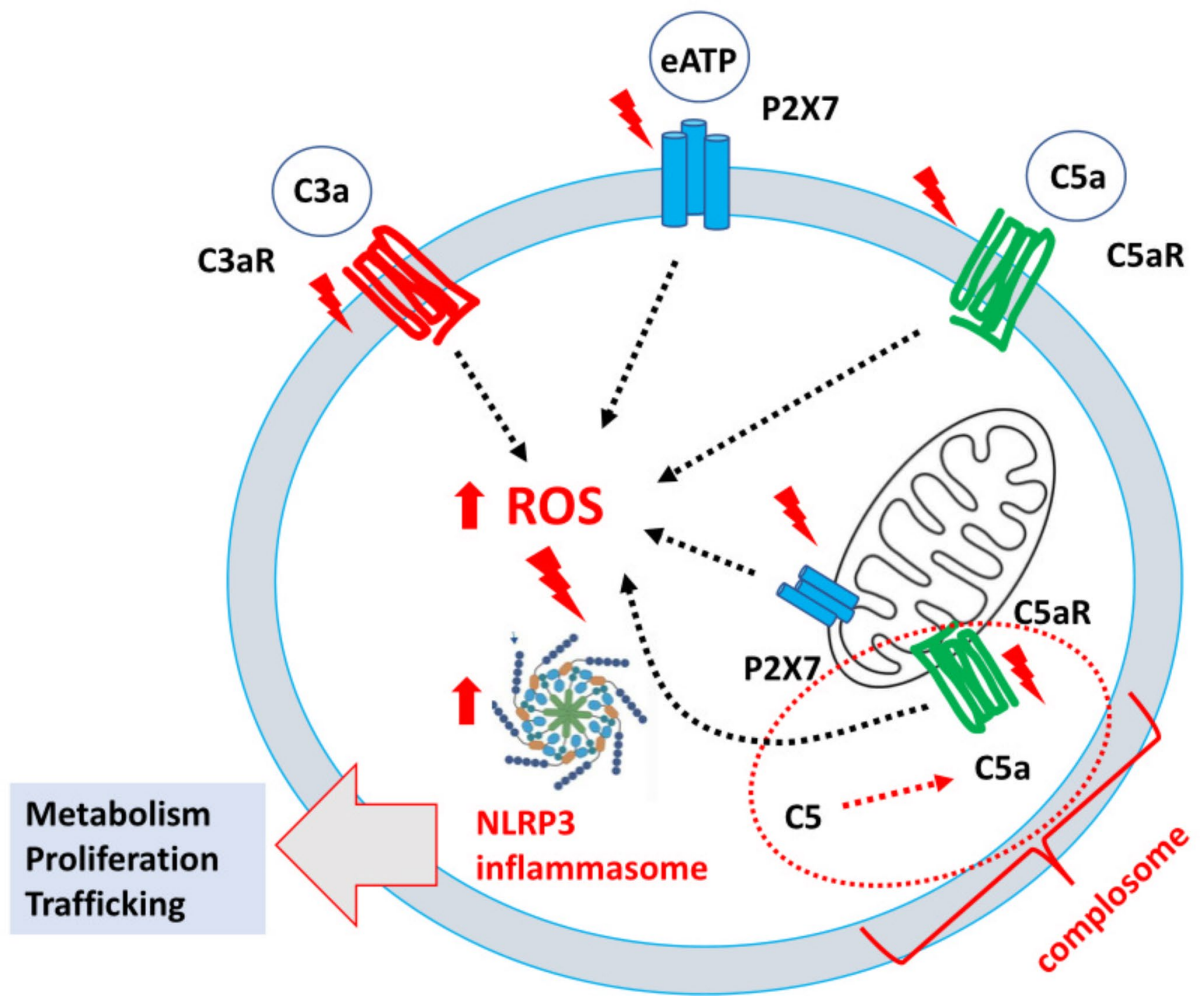
Nox2-ROS-NLRP3 inflammasome axis regulates HSPC biology. Intracellular ROS in HSPCs is generated by hematopoietic cells, specifically Nox-2, in response to stimulation by peripheral blood-derived C3a, C5a or P2 × 7 receptors. Peripheral blood-derived C3a, C5a and eATP activate C3aR, C5aR and P2 × 7 receptors on the outer cell membrane, and complosome-derived intracellular C5 a activates C5aR expressed on the mitochondrial membrane (dotted red ellipse). Mitochondria expressed P2 × 7 receptors may be also activated intracellularly by eATP. ROS generated in the cytosol activate the Nlrp3 inflammasome that, within a non-toxic to the cell, “hormetic zone of activation,” enhance the metabolism, proliferation and migration of HSPCs.

We postulate that the response of HSPC to stress is orchestrated by the coordinated activation of purinergic signaling and the ComC [13]. We suggested that the effects of both pathways merge on the activation of the intracellular nitric oxide synthetase (Nox2)-reactive oxygen species (ROS)-NLRP3 inflammasome axis. As mentioned above, activation of P2X receptors by eATP and the C3a receptor (C3aR) and C5a receptor (C5aR) by ComC fragments C3a and C5a, respectively, activates the NLRP3 inflammasome [27, 32]. This activation occurs because P2X and ComC receptors are associated with the Nox2 complex that elevates ROS levels in the cytosol (Fig. 2). This elevation has important metabolic consequences. To support this notion, our recent evidence demonstrated that the Nox2-ROS-NLRP3 inflammasome axis regulates the metabolism of HSPC and provides structural lipid elements for the formation of membrane lipid rafts (MLR) [32]. These nanoscale glycoprotein microdomains range from 10 to 200 nm in size, are enriched in cholesterol and sphingolipids floating freely in the cell’s outer membrane, optimize the response of HSPCs to external stimuli, and provide a novel level of regulation and integration of microenvironmental signals that translate into a cell response [26, 33, 34]. At the molecular level, MLR assemble cytosolic signaling molecules with cell surface “raftophilic” receptors for growth factors, cytokines, chemokines, bioactive lipids, extracellular signaling nucleotides, and adhesion molecules [33]. Thus, MLR could be considered sorting hubs that orchestrate optimal signaling through assembly of cell surface receptors and signaling proteins [33, 34].

Our recent data revealed that activation of the Nox2-ROS-NLRP3 inflammasome axis in HSPC triggers the pentose phosphate cycle, a crucial intracellular pathway that generates the cofactor nicotinamide adenine dinucleotide phosphate (NADPH); NADPH is then used in anabolic reactions in cells, including the synthesis of structural cholesterol and lipid components of MLR [32, 35]. We noticed that stimulation of HSPC by eATP as well as C3a, and C5a increases expression of mRNA for crucial regulators involved in lipogenesis; specifically, we observed increased expression of mRNA for (i) SREBP2, a ubiquitously expressed transcription factor that controls cholesterol homeostasis by stimulating the transcription of sterol-regulated genes, (ii) HMGCR, the rate-controlling enzyme in the mevalonate pathway that produces cholesterol and other isoprenoids, (iii) HMGCS, which catalyzes the reaction in which acetyl-CoA condenses with acetoacetyl-CoA to form 3-hydroxy-3-methylglutaryl-CoA (HMG-CoA), an intermediate in both cholesterol synthesis and ketogenesis, and (iv) ASMase, an enzyme responsible for catalyzing the breakdown of sphingomyelin to ceramide and phosphorylcholine [32]. The final effect of MLR formation in HSPCs stimulated by C3a, C5a and eATP was subsequently confirmed by visualization of MLR formation on outer cell membranes by confocal microscopy and Western blot analysis [32].

In addition to changes in the expression of enzymes involved in lipogenesis, we also noticed increased expression of some enzymes involved in glycolysis and protein synthesis [32]. Thus, stimulation of HSPC by eATP, C3a, and C5a provides both signals for MLR formation and stimulates metabolism in these cells to supply their energy needs in situations of stress. Further evidence of Nox2-ROS-NLRP3 inflammasome axis involvement in these metabolic changes was shown in experiments where this axis had been blocked through inhibition of the NLRP3 inflammasome by the small molecular inhibitor MCC950, as well as from analysis of Nox2-KO animals [32].

## Future implications for regulating the Nox2-ROS-NLRP3 inflammasome axis

The response to inflammatory factors as depicted in Fig. 2 may have either beneficial or detrimental effects for HSPC. This effect depends on the intracellular level of ROS activation. With a low level of ROS activation, within the so called safe “hormetic zone” [36, 37], eATP, C3a and C5a may facilitate cell metabolism, migration, and energy supply. In contrast, elevated levels of ROS cause cell damage by pyroptosis and may lead to cell aging and malignant transformation.

These dual effects of Nox2-ROS-NLRP3 may be subject to pharmacological modifications. Moderate activation of this axis may be beneficial to enhance mobilization of HSPC in order to harvest enough of these cells for transplantation during pharmacological mobilization [27]. Furthermore, since eAdo inhibits this effect by activating the A2B receptor, prevention of the inhibitory effects of eAdo generation by impairment of CD39 and CD73 ectonucleotidases or blockage of the A2B receptor may have a positive effect on increasing the number of harvested HSPC [11, 38]. This effect may also be achieved through direct stimulation of the Nox2-ROS-NLRP3 axis in harvested HSPC before transplantation, as this may facilitate their homing and subsequent engraftment. This could be achieved through ex vivo exposure of to-be-transplanted cells to eATP, C3a or C5a [32] or by prevention of the inhibitory effects of eAdo using A2B receptor blocking agents [11, 24, 32]. The NLRP3 inflammasome may also be directly stimulated through exposure of HSPCs to nigericin, a nontoxic antibiotic and potent activator of the NLRP3 inflammasome [39].

## Conclusion

The coordinated action of purinergic signaling and the ComC regulates the biology of HSPCs. Both pathways respond to stressors, activate each other in a reciprocal manner, and merge on the regulation of the NLRP3 inflammasome, a cytosolic pattern recognition receptor. Activation of the NLRP3 inflammasome, depending on activation level, may be beneficial or detrimental to HSPCs. Currently, small molecular modifiers of both purinergic signaling and ComC pathways are available that allow control of these effects.

## Data Availability

Detailed data are available upon request. **Legends to the Figures**.
